# Direct determination of cadaverine in the volatile fraction of aerobically stored chicken breast samples

**DOI:** 10.1007/s00706-018-2218-7

**Published:** 2018-08-07

**Authors:** Wojciech Wojnowski, Justyna Płotka-Wasylka, Kaja Kalinowska, Tomasz Majchrzak, Tomasz Dymerski, Piotr Szweda, Jacek Namieśnik

**Affiliations:** 1 Department of Analytical Chemistry, Gdańsk University of Technology, Gdańsk, Poland; 2 Department of Pharmaceutical Technology and Biochemistry, Gdańsk University of Technology, Gdańsk, Poland

**Keywords:** Amines, Extraction, Gas chromatography, Green chemistry, Mass spectroscopy, Proteins, Solvent-free

## Abstract

**Abstract:**

To supplement the currently used methods for poultry meat shelf life assessment, it might be necessary to develop a technique for rapid headspace analysis of volatiles with no prior sample preparation step. Biogenic amines, in particular cadaverine, are considered meat spoilage indicators. Described in this article are the results of a preliminary investigation of the applicability of proton transfer reaction mass spectrometry in the determination of cadaverine concentration in the volatile fraction of poultry meat samples stored in aerobic conditions. Dispersive liquid–liquid microextraction–gas chromatography–mass spectrometry and determination of total viable bacteria were used as reference methods. It was determined that there is a good correlation (Pearson correlation of 0.96) between the concentration of cadaverine in the headspace of chicken meat samples stored over a period of 5 days and the total viable bacteria count. Based on the results, it can be concluded that the changes of cadaverine concentration in the meat samples’ volatile fraction can be successfully monitored with a short time of a single analysis and with no sample preparation.

**Graphical abstract:**

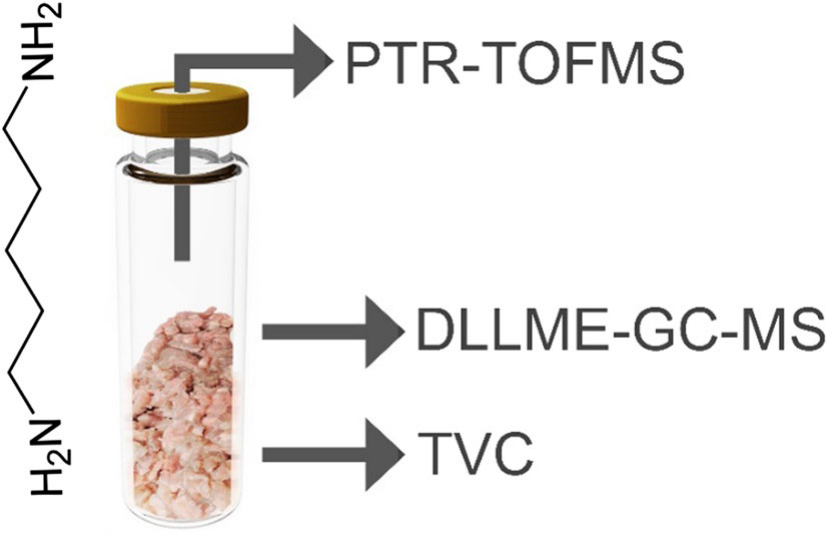

## Introduction

Facilities and plants in which poultry meat is being processed are obligated to determine its shelf life. The total viable bacteria count (TVC) is the method most widely used for this type of determination [1]. However, it should be noted that the shelf life determined based on this method pertains to batches which have already entered distribution, and the shelf life declared on the product packaging is a prognosis based on the previous measurements. Thus, any irregularities can only be detected retrospectively. Moreover, the TVC counts indicate mostly the number of bacteria which proliferate in room temperatures—the mesophilic bacteria, and not of psychrotrophic bacteria that are better adapted to low temperatures [2].

An alternative to the time-consuming TVC analysis would be a rapid method based on direct headspace analysis of meat samples and detection of volatile spoilage markers without the need for sample preparation. In particular, biogenic amines (BAs) that are the products of bacterial decarboxylation of amino acids can be identified in poultry meat and used as spoilage indicators [3]. In particular, the biological amines index (BAI = histamine + putrescine + cadaverine + tyramine) has been used to evaluate the freshness of meat products [4–6]. Among these, cadaverine was reported to be a reliable spoilage indicator of aerobically stored poultry meat [7]. However, since the biogenic amines are present in fresh meat and poultry at relatively low concentration levels [8], their detection and determination is not straightforward and usually requires a lengthy sample preparation step. Efforts have been made to curtail the time of the determination of cadaverine in chicken samples to as little as 2 h using HPTLC [9], but for the purpose of routine analysis in processing facilities, an even shorter time of a single analysis would be desirable. For that reason, we propose to tentatively determine the concentration of cadaverine in the headspace of poultry meat samples using proton transfer reaction mass spectrometry (PTR-MS) which would allow a rapid (< 15 min), routine analysis without the need for sample preparation. The technique has been described in detail by Jordan et al. [10].

In this work, we examined the applicability of the proton transfer reaction time-of-flight mass spectrometry for the detection of cadaverine in the volatile fraction of chicken meat samples stored in aerobic conditions. The analysis of total viable bacteria counts was used as a reference method. The presence of cadaverine and other biogenic amines in the analyzed samples was confirmed using a procedure based on dispersive liquid–liquid microextraction–gas chromatography–mass spectrometry (DLLME-GC–MS).

## Results and discussion

To confirm the presence of cadaverine and other biogenic amines in the poultry, samples were analyzed on the first (D_1_) and fifth day (D_5_) of storage using DLLME-GC–MS. The results of the determination of cadaverine (CAD), histamine (HIST), putrescine (PUT), and tyramine (TYR) are shown in Table 1.

**Table 1:** Information on the concentration level of determined BAs in chicken meat on the first and fifth day of refrigerated storage

Analyte	Mean concentration/µg/g ± standard deviation	
	D_1_	D_5_
CAD	n.d.	8.9 ± 1.9
HIST	1.61 ± 0.53	4.41 ± 0.47
PUT	1.019 ± 0.042	1.173 ± 0.044
TYR	n.d.	3.14 ± 0.27

The concentration of the analyzed BAs was higher after 5 days of storage in aerobic conditions. In particular, CAD, HIS, and TYR levels increased from below the LOD to 8.9 ± 1.9, 4.41 ± 0.47, and 3.14 ± 0.27, respectively. The concentration of PUT also increased during storage, albeit not significantly. The proposed methodology is robust and reliable; however, it requires time-consuming sample preparation as well as the use of standards.

At the same time, the headspace of nine samples was analyzed daily using PTR-MS to the total of 45 samples. From the four BAs, only the concentration of cadaverine exceeded the LOQ. This is in line with the GC–MS measurements and suggests that HIST, PUT, and TYR are not sufficiently abundant and/or volatile to be currently used as a reliable indicator of shelf life based solely on the analysis of the sample’s volatile fraction. However, the results of cadaverine determination correlated well with the results of total viable aerobic bacteria counts (Pearson correlation of 0.96), as shown in Table 2. Both the TVB count and the concentration of CAD in the sample’s headspace increased markedly after the second day of storage which is consistent with prior literature reports [11, 12].

**Table 2:** The concentration of cadaverine (*m/z *= 103) in the samples’ headspace (ppbv) ± intra-sample (*n* = 9) standard deviation and the total viable aerobic bacteria count (Log5 CFU/g) ± intra-sample standard deviation during five consecutive days of refrigerated storage

	Day of refrigerated storage				
	D_1_	D_2_	D_3_	D_4_	D_5_
CAD	n.d.	0.407 ± 0.050	0.99 ± 0.26	1.97 ± 0.36	2.90 ± 0.54
TVB	1.03 ± 0.29	1.02 ± 0.13	5.99 ± 1.57	8.67 ± 2.19	10.3 ± 4.2

Based on the obtained results, it can be assumed that proton transfer reaction mass spectrometry can be successfully used to monitor the concentration of cadaverine in the headspace of poultry meat samples with no prior sample preparation step. It should be noted, however, that the concentration of CAD in the samples volatile fraction is at the low ppbv level and more than three degrees of magnitude lower than in the sample itself. This means that very sensitive headspace analysis techniques such as PTR-TOFMS are needed for its reliable determination which might be an obstacle in its use as a spoilage indicator in routine analysis at the industrial level. Moreover, using PTR-MS with no prior sample preparation it was not possible to quantify HIST, PUT, and TYR, and thus to determine the value of the BAI which is a more robust meat freshness indicator than just CAD due to the complex nature of the metabolic processes of various aerobic bacteria [13].

## Conclusion

The current gold standards in the evaluation of poultry meat freshness are relatively time-consuming. A technique based on rapid headspace analysis with no prior sample preparation step could be used to supplement the currently used methods, especially in meat processing plants. Biogenic amines are considered reliable spoilage indicators. However, due to their low concentration in the volatile fraction of chicken meat samples, they cannot be reliably determined using relatively inexpensive methods such as devices equipped with chemical gas sensors. Instead, proton transfer reaction mass spectrometry can be used to monitor the changes in the concentration of cadaverine in meat sample’s headspace directly and in real time, using ambient air as a carrier gas. The technique could be developed into a dedicated commercial application for large processing plants and QA/QC laboratories. However, at the current stage of development, the technique is likely too costly and not sufficiently portable to be utilized at the distribution and retail level. The presented research is a part of a preliminary study. After verifying that PTR-TOFMS can be successfully used direct monitoring of changes in cadaverine concentration in poultry meat’s volatile fraction, it is necessary to establish the robustness of such shelf life assessment method. The variables which might impact the correlation of CAD and TVB scores are, among others, the rearing conditions of chicken, sanitary conditions during processing and distribution, and also the storage temperature and conditions. In this study, changes in the headspace of the aerobically stored product were investigated; however, an increasing proportion of meat products is being distributed and stored in modified atmosphere packaging which inhibits the growth of aerobic bacteria.

## Experimental

### Reagents and standards

Chloroform, pyridine, isobutyl chloroformate (ICBF), biogenic amine standards (cadaverine, histamine, putrescine, tyramine), and internal standard (hexylamine) were obtained, mostly as hydrochloride salts, from Sigma-Aldrich (Steinheim, Germany). Ultrapure water was obtained from a Milli-Q water purification system (Millipore, Bedford, MA). The amine standard solutions (1.0 mg/cm^3^) were prepared individually by dissolving the pure compounds in deionized water. Concentrated solutions of amine standards were prepared by diluting the standard solution with water. The solutions were stored at 4 °C in silanized screw-capped vials with solid PTFE-lined caps (Supelco, Bellefonte, PA).

### Samples

Samples of ground chicken (Gallus domesticus) breast meat were obtained from the local distribution centres in Gdańsk, Poland. The chickens were slaughtered and the carcasses were processed and transported under refrigeration to the distribution centres in the evening prior to the first day of the analysis, where they were kept at 2.4 °C. On the first day of the analysis, 0.5 kg of chicken breast was ground and transported to the laboratory in a sterile, portable refrigerator within 0.5 h. Samples of 4 g each were then placed in 20 cm^3^ glass headspace vials, covered with food wrap to emulate real storage conditions of fresh meat and refrigerated at 4 °C. The measurements were carried out for 5 consecutive days, since it was determined in an earlier study that beyond that point samples of aerobically stored chicken meat are deemed unacceptable by the consumers based on olfactory analysis [14].

### GC–MS sample preparation and analysis

A procedure based on dispersive liquid–liquid microextraction–gas chromatography–mass spectrometry (DLLME–GC–MS) was applied to determine the biogenic amines in meat samples. One gram of meat sample was placed in 20 cm^3^ screw cap vials and spiked with IS (50 mm^3^ of a water solution containing the internal standard at 100 mg/dm^3^). A mixture of methanol (600 mm^3^), pyridine:HCl (1:1 v/v) and isobutyl chloroformate (200 mm^3^) was rapidly injected into the sample tube. After 10 min, 1 cm^3^ of chloroform was added and shaken by hand for 5 min. 200 mm^3^ of the bottom layer was taken for further analysis performed using GC–MS. The gas chromatograph 7890A (Agilent Technologies) equipped with an electronically controlled split/splitless injection port was interfaced to an inert mass selective detector (5975C, Agilent Technologies) with EI ionization chamber. GC separation was performed on ZB-5MS capillary column (30 m × 0.25 mm I.D., 0.25 µm film thickness) (Zebron Phenomenex, Torrance, CA). The injection was made in splitless mode at 240 °C. Helium was used as the carrier gas at 1.0 cm^3^/min. The oven temperature program was as follows: 45 °C held for 2 min, ramped to 160 °C at 15 °C/min and held for 2 min then ramped to 275 °C at 10 °C/min and held for 9 min. Total run time was 32 min. The MS transfer line temperature was held at 280 °C. Mass spectrometric parameters were set as follows: electron impact ionization with 70 eV energy; ion source temperature 25 °C. The MS system was routinely set in SIM mode and each analyte was quantified based on peak area using one target and one or more qualifier ion(s) (Table 3). Agilent ChemStation was used for data collection and GC–MS control.

**Table 3:** Fragments, relative intensities and retention time (*t*_*R*_) of BAs obtained by application of GC–MS technique

Analytes	*m*/*z* of SIM Ions					*t*_R_
Hexylamine (IS)	146 (99.9)	130 (76.7)	128 (14.8)			8.123
Putrescine	170 (99.9)	130 (63.6)	288 (12)			12.001
Tyramine	120 (99.9)	107 (27.7)	176 (4.6)	237 (2.2)	337 (1.4)	13.509
Cadaverine	130 (79)	84 (82)	129 (73)	302 (2)		13.712
Histamine	194 (99.9)	238 (16.7)	138 (25.8)			14.324

The method’s linearity was determined by a regression analysis of the relative area (ratio of the peak area of BAs to the peak area of the IS) versus the amine concentration. Thus, 5 aqueous solutions containing all analytes with different concentration ranges were subjected to the whole analytical procedure. The results obtained showed that linearity was excellent for all the compounds with correlation coefficients (*R*^2^) ranging from 0.9934 to 0.9972. The recovery was determined by comparing unspiked meat samples to spiked meat samples at 0.15 mg/dm^3^; *n* = 4. The average recovery values ranged from 74% to 89% as shown in Table 4. The intra-day precision was determined by analyzing four replicates of meat samples spiked at 0.15 mg/dm^3^ on the same day. The relative standard deviation (RSD) for intra-day precision ranged from 2 to 4%. The limits of detection (LOD) and quantitation (LOQ) were calculated based on the ratio of 3.3 and 10 σ/S, respectively. Thus, *σ* is the standard deviation of the response, and S is the slope of the calibration curve. The LODs ranged from 1.4 to 4.2 µg/dm^3^ and the LOQs ranged from 4.6 to 12.6 µg/dm^3^.

**Table 4:** Average recoveries (%), intra-day repeatability (%RSD), and limits of detection (LOD, (µg/dm^3^) and quantification (LOQ, (µg/dm^3^)) obtained with the optimized method in spiked chicken meat samples, analyzed using GC–MS (*n* = 4 at each level)

Analyte	Concentration level 0.15 mg/dm^3^		LOD/µg/dm^3^	LOQ/µg/dm^3^
	Recovery/%	Intra-day/%RSD		
CAD	82	4	1.5	4.5
HIST	74	4	4.2	12.6
PUT	88	2	1.4	4.6
TYR	89	3	3.3	9.9

### Microbiological analysis

Samples of ground chicken breast muscles were placed in 50 cm^3^ Falcon conical centrifuge tubes (Fisher Scientific, Waltham, MA). The tubes were then filled to the volume of 40 cm^3^ with a sterilized solution of 2.2% glucose (Sigma-Aldrich, Steinheim, Germany), 2.2% peptone (Sigma-Aldrich, Steinheim, Germany), and 1.1% NaCl (POCH, Gliwice, Poland) and centrifuged for 2 min. Next, 0.1 cm^3^ samples of decimal dilutions prepared in triplicate were poured on plate count agar (PC, BTL, pH 7.2). To determine the total viable bacteria counts, the plates were incubated at 30 °C for 72 h in accordance with ISO 4833:2013 [15].

### PTR-MS analysis

Glass headspace vials containing chicken meat samples were sealed with caps lined with a silicone-PTFE membrane and incubated at 30 °C in a custom-made thermostated incubator for 10 min to facilitate the transfer of the analytes to the headspace. The samples’ volatile fraction was analyzed using the PTR TOF 1000 Ultra (Ionicon GmbH, Innsbruck, Austria) proton transfer reaction mass spectrometer with time-of-flight analyser. Ambient air passed through an activated carbon filter (Supelpure HC, Supleco) was used as carrier gas, and it was passed through the samples’ headspace and through a transfer line heated to 70 °C at 5 cm^3^ min^−1^ into the PTR device in a dynamic headspace sampling (DHS) mode [16]. The mass spectra were registered over *m*/*z* ratios of 30–250 Da once every second for 1 min. Prior to each analysis, background noise was registered by passing the carrier gas through an empty vial. The E/N ratio was set to 130 Td and the drift chamber voltage was 520 V. IoniTOF v. 2.4.40 software was used to record the spectra and PTR-MS Viewer v. 3.2.3.0 was used to process the data. Compounds were tentatively identified based on the protonated mass and fragmentation patterns. The limit of quantification was ten times the signal to noise ratio. The proton transfer reaction rate constant of 2.0 × 10^−9^ cm^3^ s^−1^ was used for quantitative analysis.

## References

[1] El Barbri N, Llobet E, El Bari N, Correig X, Bouchikhi B (2008). Sensors.

[2] Wojnowski W, Majchrzak T, Dymerski T, Gębicki J, Namieśnik J (2017). Meat Sci.

[3] Sayem N, Daher E, Simard RE (1985). J Food Prot.

[4] Yang D, Lu A, Ren D, Wang J (2017). Infrared Phys Technol.

[5] Silva CM, Glória MBA (2002). Food Chem.

[6] Li M, Tian L, Zhao G, Zhang Q, Gao X, Huang X, Sun L (2014). Meat Sci.

[7] Balamatsia CC, Paleologos EK, Kontominas MG, Savvaidis IN (2006). Antonie Van Leeuwenhoek.

[8] Rokka M, Eerola S, Smolander M, Alakomi H-L, Ahvenainen R (2004). Food Control.

[9] Kumudavally KV, Shobha A, Vasundhara TS, Radhakrishna K (2001). Meat Sci.

[10] Jordan A, Haidacher S, Hanel G, Hartungen E, Märk L, Seehauser H, Schottkowsky R, Sulzer P, Märk TD (2009). Int J Mass Spectrom.

[11] Mayr D, Margesin R, Schinner F, Märk TD (2003). Int J Mass Spectrom.

[12] Jimenez SM, Salsi MS, Tiburzi MC, Rafaghelli RC, Tessi MA, Coutaz VR (1997). J Appl Microbiol.

[13] Halász A, Baráth Á, Simon-Sarkadi L, Holzapfel W (1994). Trends Food Sci Technol.

[14] Wojnowski W, Majchrzak T, Dymerski T, Gębicki J, Namieśnik J (2017). Monatsh Chem.

[15] International Organization for Standardization (2013) ISO Standard 4833-2:2013 Microbiology of the food chain—horizontal method for the enumeration of microorganisms—Part 2: Colony count at 30 degrees C by the surface plating technique. https://www.iso.org/standard/59509.html

[16] Wojnowski W, Majchrzak T, Dymerski T, Gębicki J, Namieśnik J (2017). J AOAC Int.

